# Dietary Pectin from *Premna microphylla* Turcz Leaves Prevents Obesity by Regulating Gut Microbiota and Lipid Metabolism in Mice Fed High-Fat Diet

**DOI:** 10.3390/foods13142248

**Published:** 2024-07-17

**Authors:** Jiaobei Gao, Mengxue Zhang, Li Zhang, Nan Wang, Yan Zhao, Daoyuan Ren, Xingbin Yang

**Affiliations:** 1 Shaanxi Engineering Laboratory for Food Green Processing and Safety Control, and Shaanxi Key Laboratory for Hazard Factors Assessment in Processing and Storage of Agricultural Products, College of Food Engineering and Nutritional Science, Shaanxi Normal University, Xi’an 710119, China; 18166690913@163.com (J.G.); dyren@snnu.edu.cn (D.R.); 2 Key Laboratory of Ministry of Education for Medicinal Resource and Natural Pharmaceutical Chemistry, College of Life Sciences, Shaanxi Normal University, Xi’an 710119, China; zmx18392669571@163.com (M.Z.); 18292606003@163.com (L.Z.); wangnan_vip@yeah.net (N.W.); yanzhao@snnu.edu.cn (Y.Z.)

**Keywords:** *Premna microphylla* Turcz, pectin, obesity, fat thermogenesis, gut microbiota

## Abstract

The present study was designed to investigate the protective effects of pectin extracted from *Premna microphylla* Turcz leaves (PTP) against high-fat-diet (HFD)-induced lipid metabolism disorders and gut microbiota dysbiosis in obese mice. PTP was made using the acid extraction method, and it was found to be an acidic pectin that had relative mole percentages of 32.1%, 29.2%, and 26.2% for galacturonic acid, arabinose, and galactose, respectively. The administration of PTP in C57BL/6J mice inhibited the HFD-induced abnormal weight gain, visceral obesity, and dyslipidemia, and also improved insulin sensitivity, as revealed by the improved insulin tolerance and the decreased glucose levels during an insulin sensitivity test. These effects were linked to increased energy expenditure, as demonstrated by the upregulation of thermogenesis-related protein UCP1 expression in the brown adipose tissue (BAT) of PTP-treated mice. 16S rRNA gene sequencing revealed that PTP dramatically improved the HFD-induced gut dysbiosis by lowering the ratio of *Firmicutes* to *Bacteroidetes* and the quantity of potentially harmful bacteria. These findings may provide a theoretical basis for us to understand the functions and usages of PTP in alleviating obesity.

## 1. Introduction

Recently, obesity has been an increasing social and health problem with the booming of the economy [1,2,3]. Generally, obesity is defined by increased body weight, adipose tissue buildup, insulin resistance, hypertrophy of adipocytes, metabolic endotoxemia, persistent inflammation, and dysbiosis of the gut microbiota; thus, its effective treatment has also been essential for maintaining the health of the public [4,5]. The major goal of the current anti-adiposis tactics is to limit the intake and consumption of energy [6]. However, the issue of obesity is still far from being satisfactorily addressed.

Recent research has demonstrated that upregulating thermogenesis in adipose tissue can help treat obesity [7]. Therefore, efforts to expend excess stored energy by generating heat have been deemed an ideal strategy against obesity without changing energy intake. Adaptive thermogenesis refers to the generation of heat by the body in response to extraneous stimulation, which is separated into shivering and non-shivering thermogenesis (NST), and this process can be leveraged to counteract the hypercaloric state of obesity [8]. Studies have shown that UCP1 in brown adipose tissue (BAT) and beige adipose tissue is capable of facilitating weight control. In this regard, the activation of fat’s thermogenesis via the upregulation of UCP1 in BAT and beige adipose tissue is effective for increasing the energy expenditure [9].

Gut microbiota are well known to play an important role in the pathogenesis of obesity, which is an important environmental factor for energy acquisition and storage [10,11,12]. Interestingly, some studies have suggested that changes in the composition of gut microbiota are related to adipose thermogenesis [13,14]. Numerous studies point out that polysaccharides, dietary fibers, and pectin can lower body weight by stimulating the growth of particular anti-obesogenic intestinal bacteria and the generation of microbiota-derived compounds or by decreasing the bacteria that contribute to overweight [15,16,17,18,19,20]. Therefore, achievable strategies for modulating the gut microbiota have been put forward to prevent obesity.

The leaves of *Premna microphylla* Turcz, a traditional Chinese herb, have been used to prepare a green Chinese traditional food, which is known locally as “Shenxian tofu” due to its long history [21]. Importantly, *P. microphylla* Turcz leaves are rich in pectin (PTP) [22], which has extensive prebiotic properties, and thus, PTP may have the potential to regulate the structure of intestinal microbiota. Nevertheless, as far as we know, there are no studies exploring the anti-obesogenic effects of PTP via gut-microbiota-controlled fat thermogenesis.

In this study, PTP as pectin was extracted from leaves of *P. microphylla* Turcz under acidic conditions, and the chemical characterization of PTP, including monosaccharide composition and surface microstructures, was investigated. Additionally, the preventive effects and underlying mechanism of PTP against obesity were studied through evaluating glucolipid metabolism, intestinal microbiota homeostasis, and fat thermogenesis in HFD-fed mice for the first time. 

## 2. Materials and Methods

### 2.1. Materials and Chemicals

The assay kits of serum total cholesterol (TC), total triglyceride (TG), adiponectin (ADPN), lipopolysaccharide (LPS), high-density lipoprotein-cholesterol (HDL-C), and low-density lipoprotein-cholesterol (LDL-C) were purchased from Nanjing Jiancheng Bioengineering Institute (Nanjing, China). UCP1 antibody (ab10983) was obtained from Abcam (Cambridge, MA, USA), and anti-rabbit second antibody (KIT530) was obtained from Maixin (Maixin, Fuzhou, China). Triethylamine (TEA) and 1-phenyl-3-methyl-5-pyrazolone (PMP) were products from Merck (Darmstadt, Germany). D-Mannose, D-ribose, L-rhamnose, D-galacturonic acid, D-glucuronic acid, D-glucose, D-galactose, and L-arabinose were obtained from Sigma (Sigma-Aldrich GmbH, Sternheim, Germany). Deionized water was prepared by using a Millipore Milli Q-Plus system (Millipore, Bedford, MA, USA). Other reagents or standards were bought from Yuanye Biotechnology Co., Ltd. (Shanghai, China). All other reagents and chemicals were of analytical grade. 

### 2.2. Extraction and Purification of Pectin

The extraction of pectin from *P. microphylla* Turcz leaves (PTP) was based on the method described by previous studies with some modifications [23,24]. In brief, *P. microphylla* Turcz leaves were shattered by a pulverizer and sieved by 80 mesh to obtain the powders. The dry powder was mixed with 95% ethanol (1:10, *w*/*v*) and stirred for 4 h to remove fat-soluble impurities, and the operation was repeated three times. The residues were extracted with hydrochloric acid aqueous solution (1:10, *w*/*v* and pH = 2.0) over two cycles at 86 °C for 3.5 h, the extracts were centrifuged at 4000× *g* for 15 min, and then the combined supernatant was concentrated to one-third of the original amount at 60 °C under reduced pressure conditions. The obtained product was slowly added to the triploid volume of anhydrous ethanol by constant stirring and was kept overnight at 4 °C, and pectin precipitate was obtained by filtering. The separated pectin precipitate was dissolved in distilled water and deproteinated with sevage reagent (CHCl_3_:BuOH = 5:2, *v*/*v*) 5 times. Ultimately, the pectin from *P. microphylla* Turcz leaves (PTP) was dialyzed with distilled water for 48 h (cut-off Mw 8000 Da) before lyophilization for further study.

### 2.3. Chemical Composition of PTP

The carbohydrate content of PTP as pectin was determined by carbazole colorimetry using high-purity galacturonic acid (GalA) as a standard [25]. In brief, 5 mL of highly concentrated sulphuric acid was added to 1 mL of 0.1 g/L pectin solution, and then 0.3 mL 0.15% carbazole in ethanolic solution was added after being cooled to room temperature. A spectrophotometer was used to measure absorbance at 530 nm, and a standard curve was created with absorbance as the ordinate and concentration as the abscissa. Additionally, 0.5 mg/mL PTP solution was scanned within the range of 190–400 nm on a Hitachi U-3010 UV spectrophotometer (HITICHI Company, Tokyo, Japan), and deionized water was taken as a blank control. Finally, the Folin–Ciocalteu technique was used to determine the phenolic component content of PTP [26].

### 2.4. FT-IR Analysis and Microscopic Observation

The FT-IR spectra of PTP were recorded using a Fourier transform infrared spectrometer (Tensor27, Bruker, Karlsruhe, Germany) [27]. Briefly, the pectin samples and KBr were dried at 45 °C for 8 h to ensure that the moisture was removed thoroughly. Next, approximately 10 mg PTP was mixed with dried KBr (1:100) and pressed into a small translucent tablet for measurement. The spectrum of PTP was recorded in absorbance mode from 4000 to 400 cm^−1^. In addition, scanning electron microscopy (SEM, S-3400N, HITICHI Company, Tokyo, Japan) was used to observe the microstructure of the pre-treated PTP. Specifically, samples were dried at 45 °C to remove moisture, fixed on a special metal plate for gold spraying, and then observed at various magnifications.

### 2.5. HPLC Analysis for Compositional Monosaccharides of PTP

The compositional monosaccharides of PTP were analyzed by reversed-phase high-performance liquid chromatography (HPLC) with pre-column derivatization according to our previous method with some modifications [28]. Specifically, in a 5 mL ampoule sealed under a nitrogen environment, 20 mg of IKTP was digested to liberate component monosaccharides using 2 mL of 3 M TFA at 95 °C for 8 h. The hydrolyzed IKTP solution was spiked with 0.5 M PMP methanol solution (200 μL) and 0.3 M aqueous NaOH (300 μL). The solution was allowed to react for 60 min at 70 °C before being neutralized with 300 μL of 0.3 M HCl. The resultant solution was extracted with chloroform, and the organic phase was discarded following shaking. The extraction technique was performed three times. The aqueous layer was filtered through a 0.22 μm membrane for HPLC analysis. HPLC analysis of compositional monosaccharides in PTP was performed on a C_18_ column (4.6 mm i.d. × 250 mm, 5 µm, Venusil, FL, USA) at 35 °C using a ThermoFisher Scientific (Wilmington, NC, USA) UltiMate 3000 UPLC system equipped with a UV detector fixed at 250 nm and an auto-sampler. The mobile phase A was acetonitrile, and the mobile phase B was 3.3 mM KH_2_PO_4_-3.6 mM (triethylamine) TEA buffer with 10% acetonitrile. The gradient elution protocol was as follows: 93-93-90-90-85-85-20-20-93% B using a linear shift at 0-20-21-40-41-50-54-56-59 min. The injection volume was 20 μL, and elution was performed at a flow rate of 1.0 mL/min.

### 2.6. Animal Experiments of PTP

Forty healthy four-week-old male C57BL/6J mice (weight 16~18 g) were purchased from the Experimental Animal Center of the Fourth Military Medical University (Xi’an, China). Before the formal experiment, mice were placed in an animal laboratory for a week of adaptive feeding. They were given unrestricted access to water and rodent food (which included 40% maize flour, 26% wheat flour, 10% fish meal, 10% bran, 10% bean cake, 2% mineral, 1% coarse, and 1% vitamin complex, Minqian Feed Factory, Suqian, China) in this period. Mice were housed with a temperature range from 22 °C to 25 °C, a humidity range from 50% to 60%, and 12/12 h light–dark cycle. All animal studies were conducted with the approval of the Fourth Military Medical University’s Committee on Care and Use of Laboratory Animals (SYXK-007-2007), and the experimental procedure followed the Guideline of Experimental Animal Administration published by the State Committee of Science and Technology of the People’s Republic of China. 

After a week, every mouse was split up into five groups, each consisting of eight mice, including the normal diet (ND) control group, the high-fat-diet (HFD) model group, and three PTP intervention groups. The ND mice were fed the ND for the full 12-week feeding period, whereas the mice in the other four groups were fed a high-fat diet (HFD) that included 40% rodent chow, 30% lard, 3.5% minerals, 2% fiber, 0.2% NaCl, and 22.3% casein. Within PTP-treated groups, PTP was dissolved in saline and the mice were administrated with PTP at the dosages of 100 mg/kg bw (PTP-L), 200 mg/kg bw (PTP-M), and 400 mg/kg bw (PTP-H) for 12 weeks via intragastric administration once a day. The pectins in this experiment did not have any side effects or toxic effects at the doses used, and the specific dosages chosen were harmless and reasonable by dose conversion between human and animal administration. Additionally, the ND mice together with HFD mice were given normal saline via intragastric administration. In the end, all of the mice were slaughtered to extract the livers, adipose tissues, and blood after being completely sedated with inhaled isoflurane. The hepatosomatic index (HI) is the ratio of the liver weight to the body weight, and the fat index (FI) is the ratio of the weight of adipose tissues (epididymal white adipose tissue, inguinal white adipose tissue, mesenteric adipose tissue) to the body weight of mice. Mice’s blood samples were centrifuged at 3000× *g* for 10 min to extract serum. All tissues were then kept at −80 °C for subsequent analysis.

### 2.7. Biochemical Assay for the Serum and Hepatic Lipid Metabolism

The TG, TC, LDL-C, and HDL-C levels of the serum and the liver were measured using corresponding commercial diagnostic kits, and the serum concentrations of LPS and ADPN were all determined by ELISA assay kits. All experimental operations were measured in strict accordance with the corresponding operating instructions using a microplate reader (Multiskan Go, Thermo Fisher Scientific, Wilmington, NC, USA).

### 2.8. Oral Glucose Tolerance Test (OGTT) and Insulin Sensitivity Test (IST)

The mice were fasted for 10 h before the oral glucose tolerance test (OGTT) and insulin sensitivity test (IST). Oral glucose (2.0 g/kg) or intraperitoneal injection of insulin (0.625 U/kg) was carried out using a OneTouch Ultra glucometer (Lifescan Benelux, Beerse, Belgium) [29]. Blood glucose levels were monitored using the glucometer at 0, 15, 30, 60, 90, and 120 min after oral glucose or insulin injection for the OGTT and IST, respectively.

### 2.9. 16S rDNA Sequencing and Bioinformatics Assay

The colonic fecal samples were pre-treated according to our previously established methodology [30]. Using 1% agarose gel electrophoresis, the extracted DNA’s relative purity was evaluated, and a NanoDrop 2000 UV–vis spectrophotometer (ThermoFisher Scientific, Wilmington, NC, USA) was used to determine the concentration. Under the previously mentioned conditions, the V3–V4 region of 16S rDNA was amplified for 30 cycles using the primers 338F and 806R [31]. After that, the products were filtered and combined before sequencing, then filtered and trimmed, and finally clustered to amplify sequence variants (ASVs) and analyzed for classification.

### 2.10. Histopathological Observation and Immunohistochemistry

Histopathology was performed on eight samples from each group, and biological samples, including liver tissues, subcutaneous fat tissues, brown fat tissues, and epididymal fat tissues, were fixed in 4% paraformaldehyde followed by embedding in paraffin, and then the tissue samples were cut out into slices (5 μm thick) for hematoxylin and eosin (H&E) staining and immunohistochemical examination. In the immunohistochemical examination, sections were incubated with 5% BSA for 60 min at room temperature and blotted overnight at 4 °C with UCP1 antibody (1:500 dilution). Three PBS washes (8 min each) were performed on the sections. The secondary antibody was incubated for 50 min at room temperature in a temperature box once the PBS solution surrounding the tissue had dried, followed by washing three times with PBS. Finally, the histopathological and immunohistochemical pictures of the analyzed tissues were examined using an Axio Imager upright microscope (Axio Imager M2, Carl ZeissAG, Jena, Germany).

### 2.11. Statistical Analysis

Prism 8.0.1 software was used for data statistical analysis, and data were expressed as the means ± standard deviation (SD). One-way analysis of variance (ANOVA) was used to determine whether there were significant differences among various groups, and a *p* value less than 0.05 was considered dramatically significant.

## 3. Results

### 3.1. Chemical Properties of PTP

**Figure 1:**
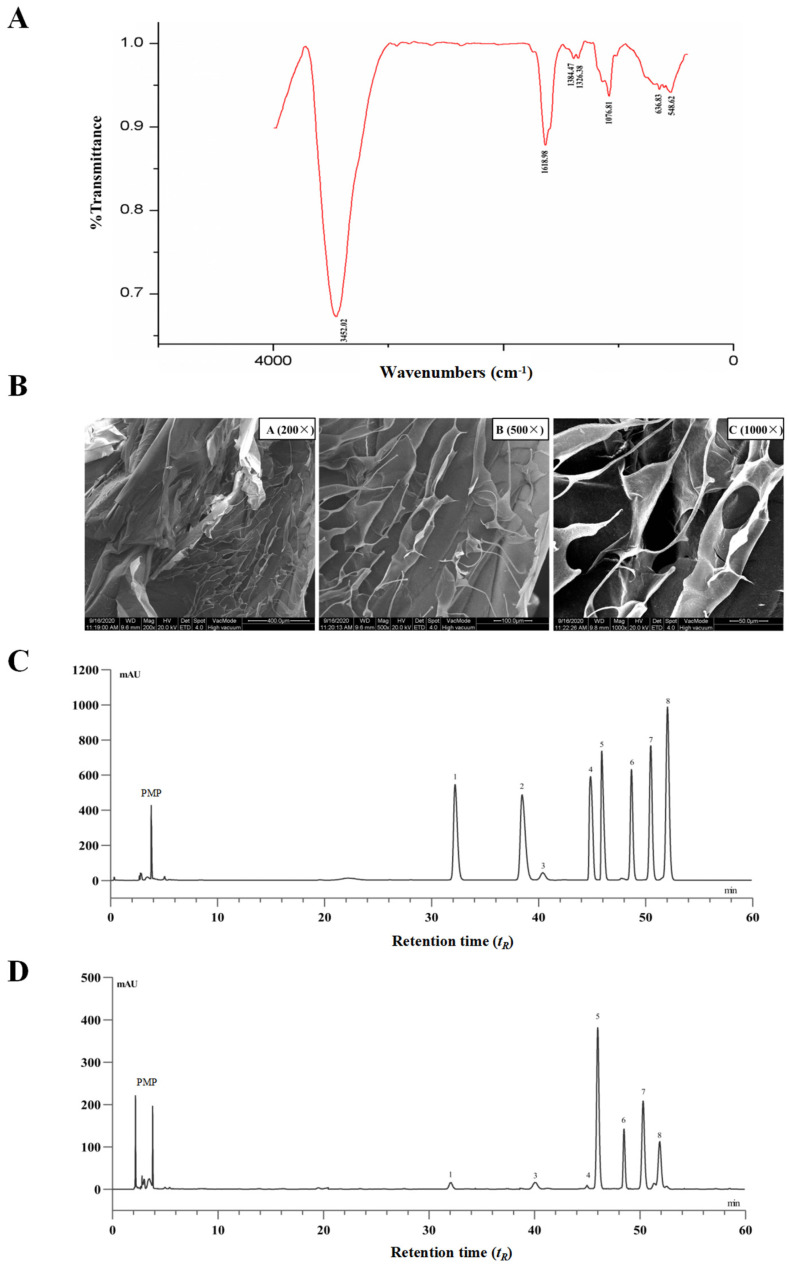
Chemical analysis of pectin from dietary *Premna microphylla* Turcz leaves (PTP). (**A**) FT-IR spectra. (**B**) Scanning electron microscope images at different magnifications. The HPLC chromatograms of PMP (1-pheny-3-methyl-5-pyrazolone) derivatives of eight standard monosaccharides (**C**) and component monosaccharides released by hydrolyzing PTP as pectin (**D**). Peaks: (1) mannose, (2) ribose, (3) rhamnose, (4) glucuronic acid, (5) galacturonic acid, (6) glucose, (7) galactose, (8) arabinose.

The extraction yield of PTP was 5.6% of dried *Premna microphylla* Turcz leaves, and the pectin content of PTP was 48.3%. The UV spectra of PTP revealed that there was no major absorption peak at approximately 260–280 nm, suggesting that there were no nucleic acids and proteins lingering in PTP. Additionally, no polyphenols and soluble reducing sugars were detected in PTP. As reflected in Figure 1A with the FTIR spectra of PTP in the region of 4000~400 cm^−1^, the characteristic absorption peaks of PTP indicated the affiliation of abundant functional groups of pectin. To be specific, the absorption peak near 3452.02 cm^−1^ was a vibration peak of the hydroxyl group. The peaks at about 1740 cm^−1^ and 1618 cm^−1^ were the GalA’s C=O stretching vibration of methyl esterified groups and ionic carboxyl groups, respectively [32]. The vibrational absorption peak at 1618.98 cm^−1^ was GalA’s ionic carboxyl group, while the peak near 1740 cm^−1^ exhibited no obvious absorption, suggesting that methyl esterification of GalA exists in the isolated pectin. The weak absorptive peak about 1384 cm^−1^ was identified as the result of O-H or C-H bending vibrations. The stretching vibrations of the C-OH side groups and the C-O-C vibration of the glycosidic bond were responsible for the absorption around 1076.81 cm^−1^, which indicated the presence of a pyranose unit in PTP as pectin and may be interpreted as the molecular fingerprint of pectins [33]. 

Here, the use of SEM at various magnifications was used to study the distinctive surface microstructure of PTP. As shown in Figure 1B, PTP was of smooth reticular structure with very close arrangement, and it was also observed that PTP had various pore structures under a high-magnification microscope, indicating that PTP possessed partial water solubility, which might be closely associated with these structural features. The surface structural properties and particle size of biopolymers have important effects on their physicochemical properties, such as their water-holding capacity, solubility, swelling, and gel formation. This phenomenon also further suggests that PTP has better water-holding capacity, for which one of the reasons may be due to the nature of the surface structure. The results of these structural characterization studies indicate that PTP is a typical pectin.

Additionally, PTP’s compositional monosaccharides were determined via HPLC. Eight PMP-derived standard monosaccharides and the tested PMP-labeled monosaccharides released from PTP samples are shown in Figure 1C and Figure 1D, respectively. By comparing the peak retention time (t_R_) of the samples with those of the monosaccharide standards, the compositional monosaccharides of PTP were identified. The most abundant monosaccharide presented in PTP was galacturonic acid with a relative molar proportion of 32.1%, and the other compositional monosaccharides in PTP were mannose, rhamnose, glucuronic acid, glucose, galactose, and arabinose in relative molar proportions of 2.3%, 1.0%, 0.73%, 8.3%, 26.2%, and 29.2%, respectively. According to these findings, PTP was a typical acidic pectin, containing the basic components galacturonic acid, glucose, galactose, and arabinose, which was in line with earlier reports and the conclusions drawn from FT-IR spectroscopy research [34] (Figure 1A).

### 3.2. PTP Prevented Obesity and Hepatic Steatosis in HFD-Fed Mice

**Figure 2:**
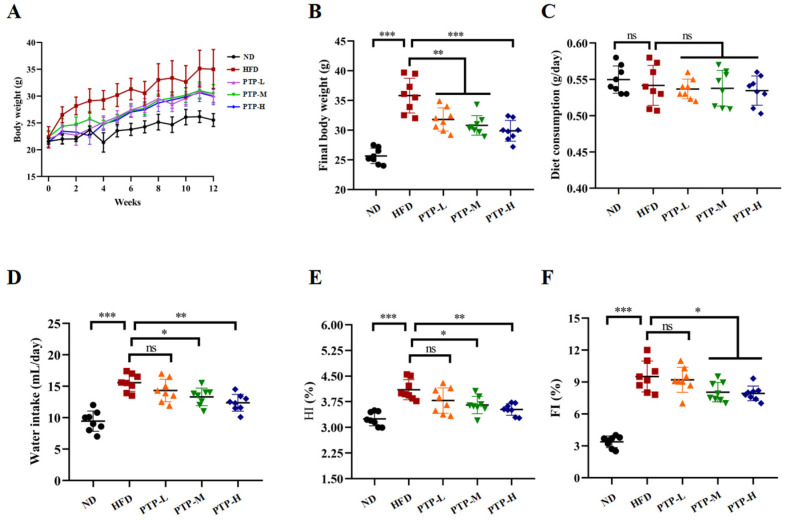
Effects of PTP on body weight, water intake, food consumption, and basic parameters for 12 consecutive weeks of high-fat diet (HFD) feeding in mice (**A**–**F**). Hepatosomatic index (HI) = liver weight (g)/body weight (g); fat index (FI) = total fat weight (g)/body weight (g); total fat weight (g) = epididymal white adipose tissue (eWAT) weight (g) + inguinal white adipose tissue (iWAT) weight (g) + mesentery adipose tissue (MAT) weight (g). One-way ANOVA followed by Tukey’s multiple comparisons test was performed for all groups. Values are expressed as means ± SD (*n* = 8). * *p* < 0.05, ** *p* < 0.01, *** *p* < 0.001 and ns indicates no significant difference.

Following a 12-week period of normal diet (ND) or high-fat-diet (HFD) feeding, Figure 2A–F shows the changes in the tested mice’s body weight, food and water consumption, hepatosomatic index (HI), and fat index (FI). Prior to the trial, the body weight of the mice in the various groups did not significantly differ from one another. However, after 12 weeks, the mice fed the HFD had a considerable increase in body weight (*p* < 0.001, Figure 2B) compared to the ND mice, proving that the obesity model was successfully created. It is interesting to note that PTP at all tested dosages significantly reduced the weight gain in HFD-fed mice (*p* < 0.01, Figure 2A,B), and the effects of PTP at dosage of 400 mg/kg bw (PTP-H) were more significant than those of the other two dosages (*p* < 0.001, Figure 2B). Additionally, the intake of foods was tested during the experiments, and there was no discernible variation between the five groups (Figure 2C). In comparison with the ND group, being fed the HFD increased the mice’s water intake (*p* < 0.001), whereas the water intake of mice in the PTP-M and PTP-H groups was reduced obviously after the administration of PTP, relative to the HFD model group (*p* < 0.05 and *p* < 0.01, Figure 2D). In addition, compared to the untreated ND mice, the mice fed a 12-week HFD showed a significant rise in the hepatosomatic index (HI) and fat index (FI) (*p* < 0.001, Figure 2E,F). However, PTP administered at 200 mg/kg bw and 400 mg/kg bw markedly reduced the HI and FI of HFD-fed mice.(*p* < 0.05, Figure 2E,F). 

**Figure 3:**
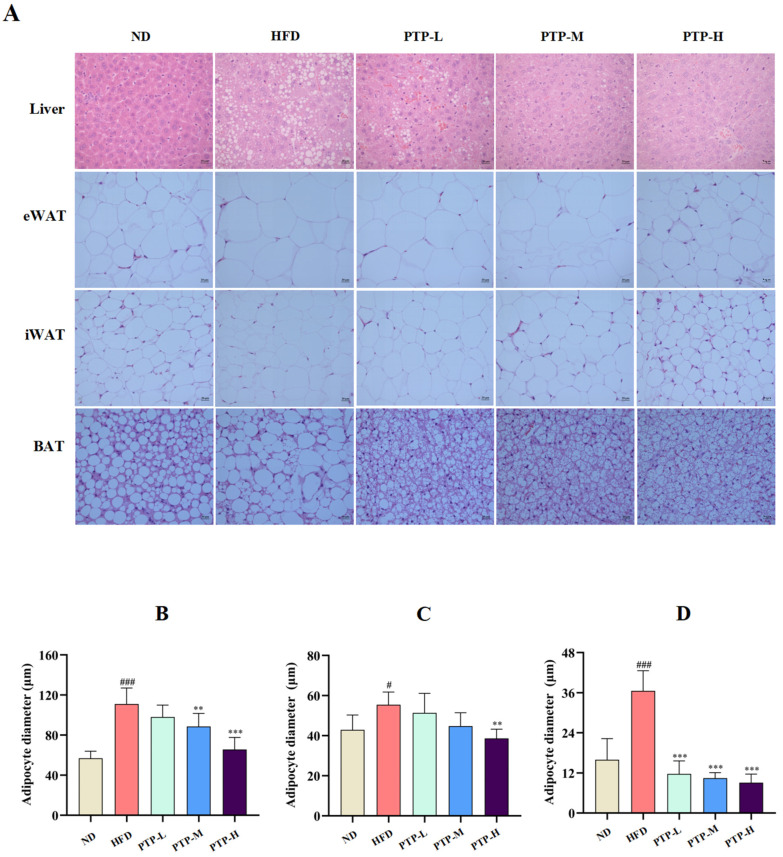
Effects of PTP on histopathological changes in hepatocytes in the liver and three different adipocytes stained with H&E, and original magnification 200×. The diameters of adipocytes were determined by ImageJ software (version 1.54) (*n* = 8 per group). (**A**) Histopathological alterations of the livers and the adipose tissues stained by H&E (original magnification of 400×). (**B**–**D**) The diameters of epididymal white adipose tissue (eWAT) and inguinal white adipose tissue (iWAT) as well as brown adipose tissue (BAT), respectively. # *p* < 0.05 and ### *p* < 0.001, versus the ND mice. ** *p* < 0.01, and *** *p* < 0.001, versus the HFD mice.

To further validate the protective benefits of PTP against HFD-induced obesity in C57BL/6J mice, histological observations of the livers and adipose tissues were performed at the end of the treatment period (12 weeks). Feeding the mice the HFD resulted in histological alterations associated with hepatic steatosis, including degeneration and vacuolation as well as abnormal fat accumulation within the liver cells or adipocytes (Figure 3A). Feeding the mice the HFD also led to larger adipocyte diameters in epididymal white adipose tissue (eWAT, *p* < 0.001, Figure 3B), inguinal white adipose tissue (iWAT, *p* < 0.05, Figure 3C), and brown adipose tissue (BAT, *p* < 0.001, Figure 3D), accompanied by fat accumulation. However, in comparison with HFD-fed mice, PTP therapy clearly reduced fat deposition in the liver and adipocytes, particularly in the PTP-H group (*p* < 0.01, Figure 3B–D). The results presented here simultaneously suggest that the proper application of PTP is efficient at inhibiting HFD-induced obesity in mice.

### 3.3. PTP Alleviated Abnormal Glucolipid Metabolism in HFD-Induced Obese Mice

**Figure 4:**
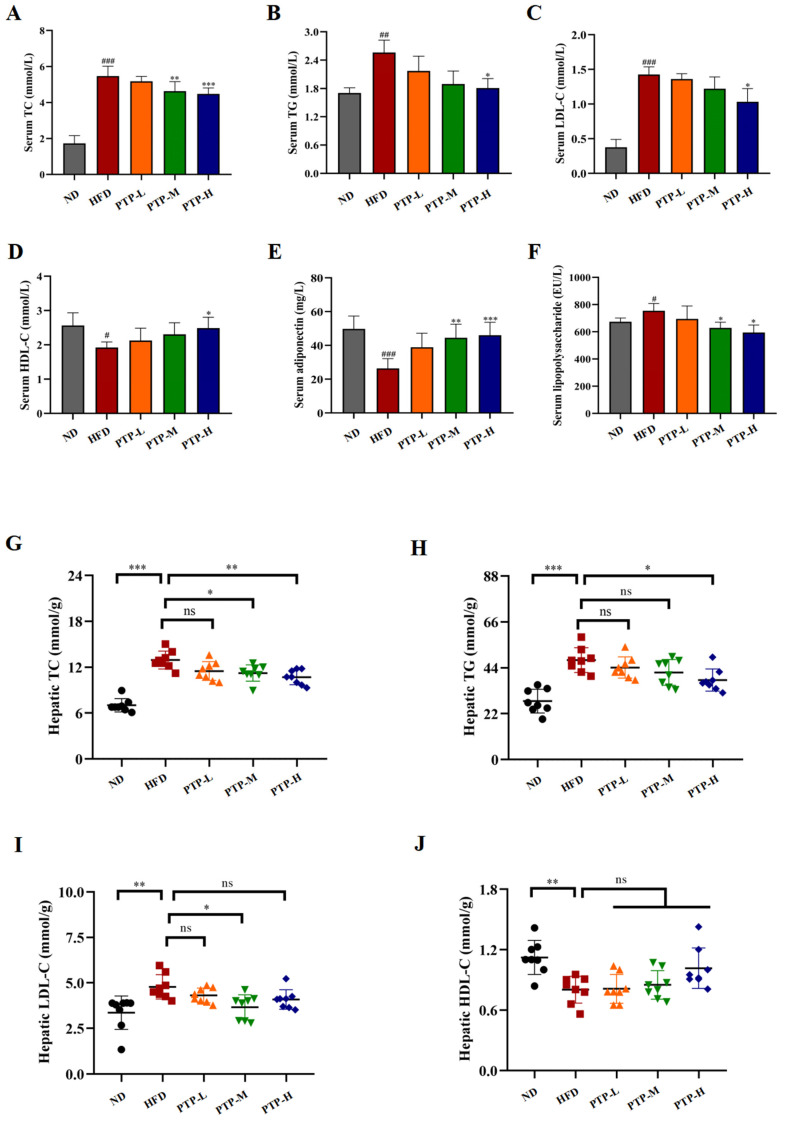
Effects of PTP on lipid metabolism and its related factors, adiponectin (ADPN) and lipopolysaccharide (LPS), in HFD-fed mice. (**A**–**F**) Serum TC, TG, LDL-C, HDL-C, ADPN, and LPS levels. (**G**–**J**) Hepatic TC, TG, LDL-C, and HDL-C levels. Data are expressed as mean ± SD (*n* = 8 per group). One-way ANOVA followed by Tukey’s multiple comparisons test were performed for all groups. # *p* < 0.05, ## *p* < 0.01, and ### *p* < 0.001, versus the ND mice. * *p* < 0.05, ** *p* < 0.01, *** *p* < 0.001 and ns indicates no significant difference, versus the HFD mice.

As shown in Figure 4, it was found that giving mice a constant 12-week HFD greatly increased the quantities of TC (*p* < 0.001), TG (*p* < 0.01), and LDL-C (*p* < 0.001) in their serum, and serum HDL-C levels decreased significantly (*p* < 0.05). Notably, PTP treatment ameliorated all these parameters or dyslipidemia in HFD-fed mice at certain levels, and more significant effects were observed in the PTP-H group (*p* < 0.05, Figure 4A–D). At the same time, the consumption of the HFD led to a notable reduction in the level of serum adiponectin (ADPN) and a striking elevation in the level of serum LPS, as well (*p* < 0.001, *p* < 0.05, Figure 4E,F). Nevertheless, PTP treatment exerted markedly protective effects against these abnormities in the serum ADPN and LPS levels caused by the HFD in comparison with the HFD model group.

In order to comprehend PTP’s impact on lipid metabolism more fully, the liver parameters related to the lipid homeostasis of the tested mice were further determined. Feeding the mice the HFD induced increases markedly in the hepatic TG, TG, and LDL-C levels, together with a marked reduced hepatic HDL-C content as compared to ND mice (Figure 4G–J). For example, the hepatic TC and TG levels of HFD-fed mice were observably increased by 85.1% and 71.4% more than those of the ND mice, respectively (*p* < 0.001). As expected, the mice administered PTP at 200 mg/kg bw and 400 mg/kg bw had a significant decrease in hepatic TC content of 18.1% and 23.2%, respectively, in contrast to the mice fed the HFD (*p* < 0.05, *p* < 0.01, Figure 4G). Additionally, compared with the HFD group, the mice in the PTP-H group displayed lower levels of TG (*p* < 0.05, Figure 4H). Moreover, the elevated hepatic LDL-L and the decreased liver HDL-L also existed in the HFD model group when compared to the ND group (*p* < 0.01, Figure 4I,J). Unexpectedly, LDL-L levels were significantly lower in mice in the PTP-M group compared to the HFD group (*p* < 0.05), whereas they were not significantly lower in mice in the PTP-H group (*p* > 0.05). Additionally, although oral administration of PTP had slight effects on hepatic HDL-L contents, none of them were significant (*p* > 0.05).

**Figure 5:**
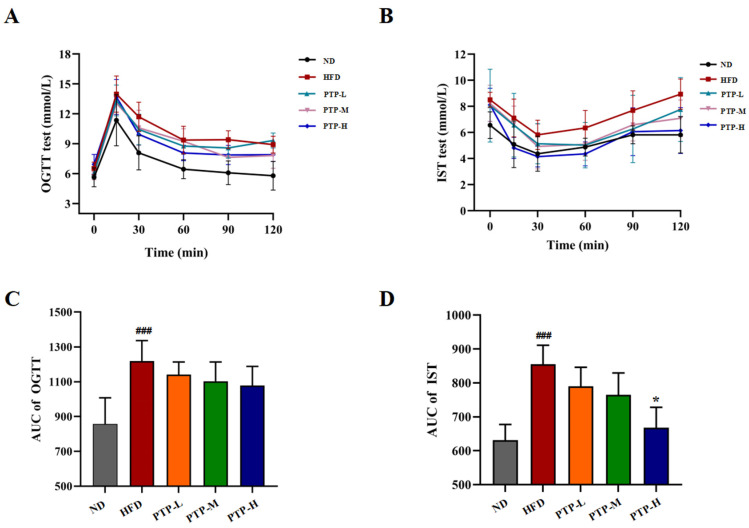
Effects of PTP on glucose tolerance (OGTT) and insulin sensibility test (IST) in HFD-fed mice. (**A**) OGTT. (**B**) IST. (**C**,**D**) Areas under the curve (AUCs) for OGTT and IST. Data are expressed as mean ± SD (*n* = 8 per group). One-way ANOVA followed by Tukey’s multiple comparisons test for were performed for all groups. ### *p* < 0.001, versus the ND mice. * *p* < 0.05, versus the HFD mice using Tukey’s multiple comparisons test for all groups. * *p* < 0.05.

Furthermore, the oral glucose tolerance test (OGTT) and insulin sensitivity test (IST) were conducted after oral glucose or insulin injection for investigating whether PTP might exert efficacy on glucose homeostasis in HFD-fed obese mice (Figure 5A,B). The area under the curve (AUC) of the OGTT and the AUC of the IST for all the tested mice were calculated by software GraphPad Prism. As displayed in Figure 5C,D, within 120 min, mice fed the HFD showed a considerably greater glycemic response to oral glucose than mice fed the ND (*p* < 0.001). The intervention of PTP reduced the AUC in HFD-fed mice, but the effects were not statistically significant (*p* > 0.05). Nevertheless, the HFD model mice exhibited insulin resistance in the IST (*p* < 0.001). It was interesting to note that the HFD-induced drop in insulin sensitivity was significantly improved by high-dose PTP treatment as reflected by the decreased glucose levels during the IST (*p* < 0.05). These findings suggest that PTP protects against insulin resistance in mice that is brought on by an HFD in mice.

### 3.4. PTP Enhanced Fat Thermogenesis in HFD-Fed Mice

**Figure 6:**
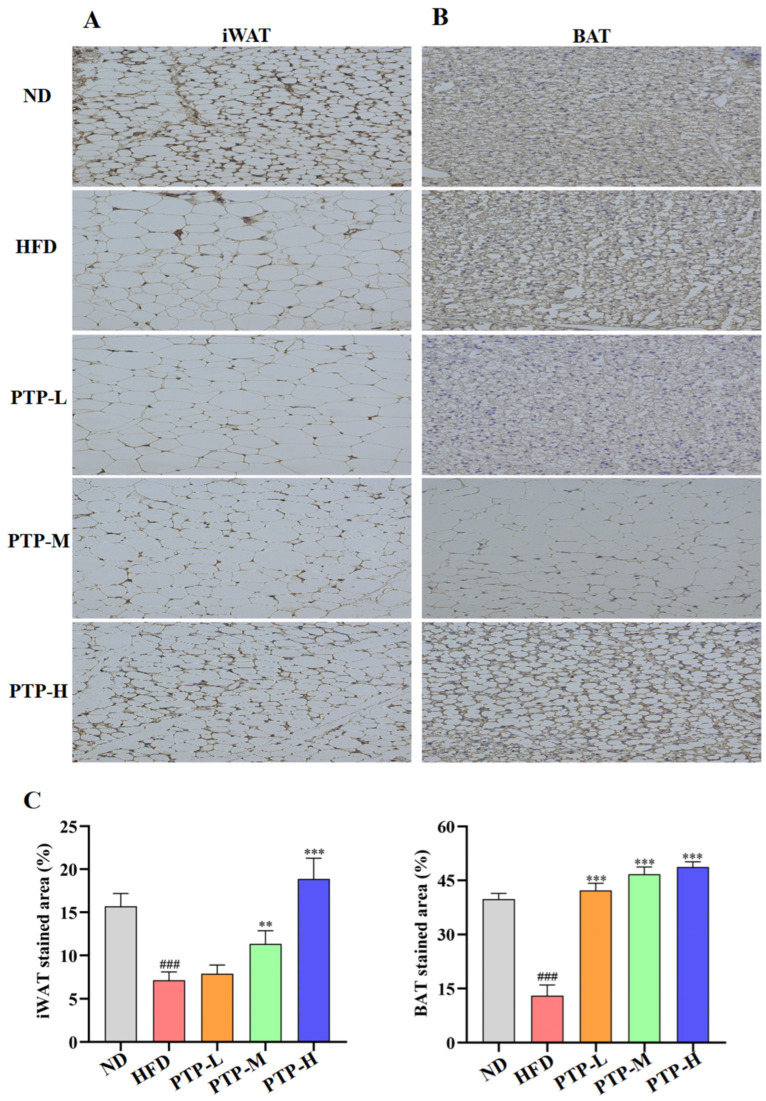
Expression of thermogenic protein UCP1 in inguinal white adipose tissue (iWAT) and brown adipose tissue (BAT), and original magnification 400×. (**A**) iWAT. (**B**) BAT. (**C**) Score of immunohistochemical analysis (*n* = 8 per group). ### *p* < 0.001, versus the ND mice. ** *p* < 0.01 and *** *p* < 0.001, versus the HFD mice.

To investigate whether the regulatory effect of PTP on lipid metabolism was related to the energy expenditure, the thermogenic protein UCP1 was expressed in iWAT and BAT and was observed by immunohistochemistry (IHC) on eight samples from each group. As described in Figure 6, the IHC-stained cells can be seen as yellow/brown positivity, and the expression of UCP1 protein in HFD-fed mice is clearly decreased in comparison to that in ND mice (*p* < 0.001 vs. ND group). However, the UCP1 protein in iWAT and BAT was remarkably enhanced in the PTP-treated mice as compared to that in HFD-fed mice (*p* < 0.001). Figure 6A,B further demonstrate the quantitative results for the IHC-stained areas of UCP1 expression in the mouse iWAT and BAT, and feeding the mice the HFD could obviously decrease the protein UCP1’s expression in iWAT and BAT (*p* < 0.001 vs. ND control). As expected, PTP treatment prominently increased the UCP1 protein expression in the iWAT and BAT of mice in comparison with the HFD-fed mice, and PTP administered at 400 mg/kg bw was more effective than the other two dosages of PTP in increasing the UCP1 expression within the iWAT of HFD-fed mice (*p* < 0.001), indicating that PTP as pectin may encourage the browning of white fat by activating BAT’s thermogenic activity. This observation indicated that PTP prevented HFD-induced obesity possibly through the activation of adipose thermogenesis to increase energy expenditure.

### 3.5. PTP Improved Gut Microbiota Dysbiosis in HFD-Fed Mice

**Figure 7:**
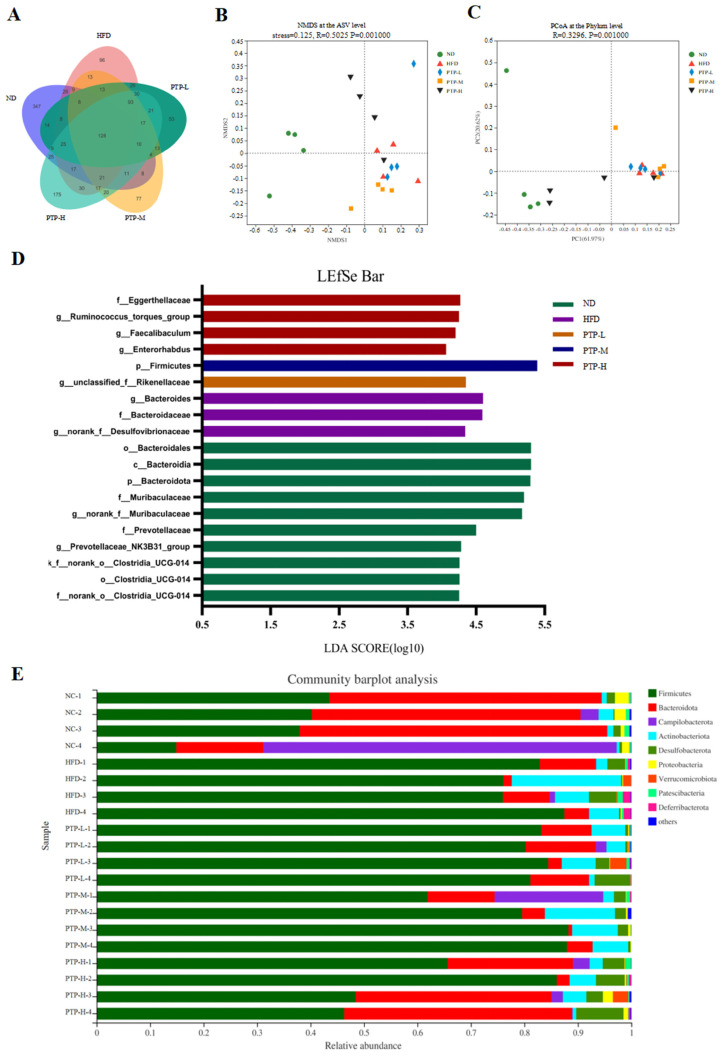
Effects of PTP on intestinal microflora structure of mice fed with HFD. (**A**) Venn diagram of bacteria detected at the ASV level. (**B**,**C**) Beta diversity analysis of intestinal microbiota using the non-metric multidimensional scaling (NMDS) and principal co-ordinates analysis (PCoA). (**D**) LEfSe analysis of microbiota. (**E**) Bacterial community at the phylum level.

**Figure 8:**
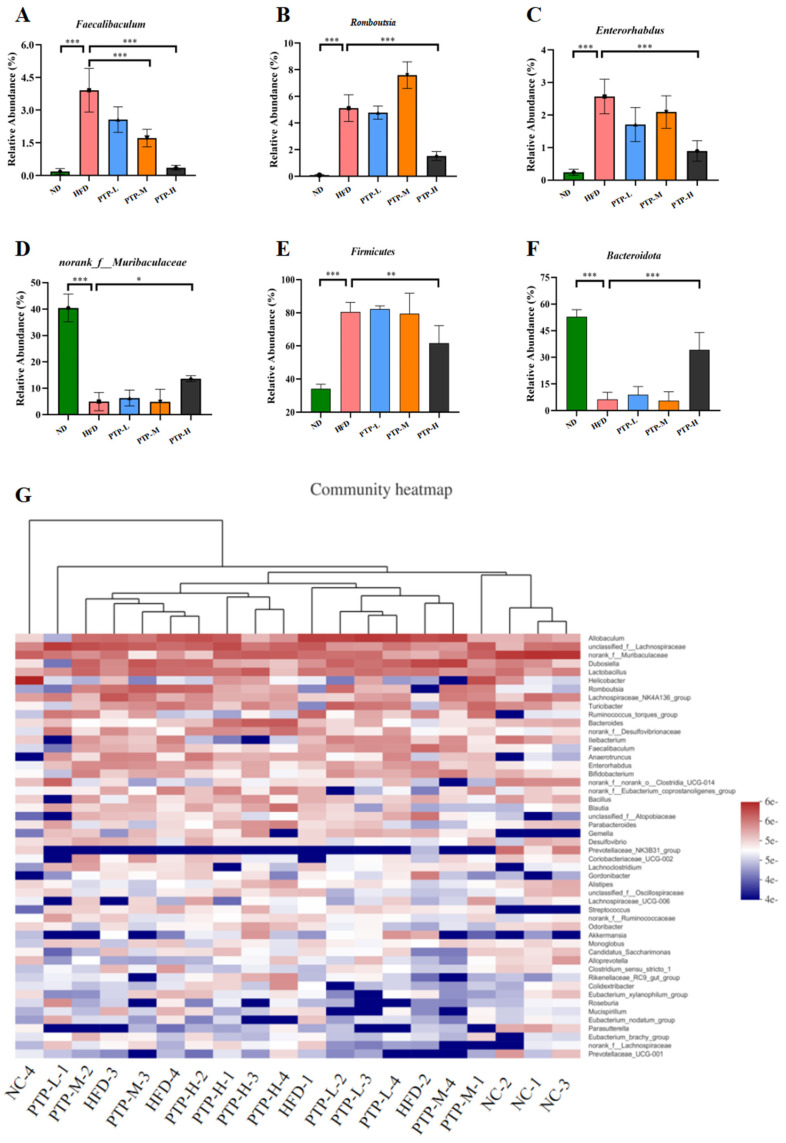
Effect of PTP on the abundance of intestinal flora in HFD-fed mice. (**A**) Relative abundance of *Faecalibaculum* at the genus level. (**B**) Relative abundance of *Romboutsia* at the genus level. (**C**) Relative abundance of *Enterorhabdus* at the genus level. (**D**) Relative abundance of *norank-f-Muribaculaceae* at the genus level. (**E**) Relative abundance of *Firmicutes* at the phylum level. (**F**) Relative abundance of *Bacteroidota* at the phylum level. (**G**) Heatmap comparison and hierarchical clustering dendrogram based on the relative abundance at the genus level. One-way ANOVA followed. * *p* < 0.05, ** *p* < 0.01 and *** *p* < 0.001, versus the HFD mice.

Accumulating evidence has revealed that the modification of the host gut microbiota is a critical influencing element in the onset of obesity. Therefore, we used 16S rRNA gene sequencing to further study PTP’s impact on gut microbiota. A total of 20 colon contents were used to generate 774,131 viable bacterial 16S rDNA gene reads, with an average of 38,706 reads per sample. A Venn diagram revealed that 128 ASVs were frequently found in each of the five groups, while the numbers of specific ASVs of the mice for the ND control, HFD model, and the three intervention groups (100, 200, and 400 mg/kg bw PTP) were 347, 96, 53, 77, and 175, respectively (Figure 7A). Meanwhile, non-metric multidimensional scaling (NMDS) at the ASV level and principal co-ordinates analysis (PCoA) at the phylum level also showed the different makeup of the microbiota in each of the five groups (Figure 7B,C). The modifications in the gut microbiota across several groups were also demonstrated by the LEfSe study (Figure 7D). At the phylum level, *Firmicutes* and *Bacteroidetes* were the two major phyla, with *Campilobacterota*, *Actinobacteriota*, and *Desulfobacterota* making up the remaining three most prominent phyla (Figure 7E). Over a 12-week feeding period, the ingestion of an HFD in mice dramatically enhanced the abundance of *Firmicutes* and reduced the abundance of *Bacteroidetes,* in contrast to the ND group (*p* < 0.001, Figure 8E,F). However, in contrast with the HFD group, the administration of 400 mg/kg bw PTP markedly decreased the *Firmicutes* abundance and notably elevated *Bacteroidetes* abundance in mice (*p* < 0.01, *p* < 0.001, Figure 8E,F). Additionally, the genus level also revealed a number of notable modifications (Figure 8G). The HFD-treated mice revealed a considerable increase in the relative abundance of *Faecalibaculum*, *Romboutsia*, and *Enterorhabdus*, and the abundance of *norank-f-Muribaculaceae* was significantly decreased (Figure 8A–D). Interestingly, the intervention of PTP inhibited the gut microbiota disturbance induced by HFD feeding, and it was found that medium and high doses of PTP decreased the abundance of *Faecalibaculum* (*p* < 0.001, Figure 8A), whereas PTP in high dose also improved dysbiosis of *Allobaculum*, *nterorhabdus*, and *norank-f-Muribaculaceae*, respectively (*p* < 0.001, *p* < 0.05, Figure 8E,F). These results suggest that PTP can regulate the balance of intestinal microorganisms in maintaining intestinal microbial homeostasis by inhibiting the reproduction of detrimental genera and promoting the growth of favorable genera in the intestine, which can improve the dysfunction of intestinal microorganisms brought on HFD feeding. Interestingly, treatment with PTP could inhibit the gut microbiota dysbacteriosis caused by HFD feeding. These results demonstrated that PTP might modify the intestinal microbial communities in HFD-induced obese mice, thereby mitigating the dysbiosis of the gut microbiota.

## 4. Discussion

Long-term HFD consumption can result in aberrant glucolipid metabolism and gut microbial dysbiosis, a type of chronic metabolic illness [35]. It has been demonstrated that certain dietary fiber supplements may influence the regulation of the gut microbiota, the prevention of inflammation, and the control of hyperlipidemia [36]. Plants contain polysaccharides, polyphenols, alkaloids, aldehydes, fatty acids, and saponins, which are believed to have the potential to enhance the body’s immune system and its antiviral and anti-inflammatory activity [37]. The fact that pectin inhibits the growth and spread of cancer cells and causes apoptosis in human cancer cells is highly intriguing as it has multiple beneficial impacts on human health [38,39], lowering cholesterol and serum glucose levels [40] and stimulating the immune response [41]. Song et al. demonstrated that putrescine pectin can ameliorate inflammation by increasing host intestinal resistance, reducing microvillus rupture in the midgut, and restoring nuclear structure of midgut cells [42]. Natural pectin is a type of dietary polysaccharide that has gained significant interest recently due to its excellent nutritional and functional value [43], and it has been demonstrated to alleviate HFD-induced nonalcoholic fatty liver diseases in mice through regulating liver lipid metabolism [44]. However, there is limited research focusing on the effects of PTP against disorders of lipid metabolism and intestinal microbiota. In this experiment, administering PTP in C57BL/6J mice inhibited the abnormal increase in body weight, visceral obesity, and dyslipidemia induced by the HFD, and also enhanced insulin sensitivity, as shown by an insulin sensitivity test’s lower glucose levels and enhanced insulin tolerance. These effects might be linked to the increased energy expenditure, as demonstrated by the upregulation of thermogenesis-related protein UCP1 expression in the brown adipose tissue (BAT) of PTP-treated mice. The 16S rRNA gene sequencing demonstrated that PTP dramatically reduced the HFD-induced gut dysbiosis by lowering the ratio of *Firmicutes* to *Bacteroidetes* and the quantity of potentially harmful bacteria, suggesting the important role of PTP in reducing metabolic injury in the livers and the adipose tissues, improving lipid and glucose metabolism, and modulating intestinal microbes.

Herein, the characteristic absorption peaks of PTP indicate the affiliation of abundant functional groups of pectin by IR spectroscopy (Figure 1), verifying the unique ionic carboxyl group (vibrational absorption peak at 1618.98 cm^−1^) of galacturonic acid in PTP. Additionally, PTP is identified as the primarily acidic pectin extracted from *P. microphylla* Turcz leaves with the compositional monosaccharides of galacturonic acid (32.1%), arabinose (29.2%), galactose (26.2%), and glucose (8.3%), measured by HPLC analysis (Figure 1), which is basically consistent with an early report [34,42]. In addition, the unique surface microstructure of PTP observed by scanning electron microscopy (SEM) at different magnifications (Figure 1) indicates that PTP is of smooth reticular structure with very close arrangement, and it is also observed that PTP has various pore structures under a high-magnification microscope, indicating that PTP is a typical pectin for possessing partial water solubility. The unique structural and chemical composition of PTP lays a theoretical foundation for exploring the group-efficacy relationship for the homeostatic regulation of glycolipid metabolism.

Furthermore, we first examined the possibility for PTP to prevent obesity caused by a high-fat diet by adjusting the gut microbiota’s composition. The present research discovered that treatment with PTP in the HFD-fed mice for 12 weeks consecutively caused decreases in body weight, the hepatosomatic index (HI), and the fat index (FI) in comparison with the HFD feeding control (Figure 2), suggesting that it was necessary to develop plant-based pectin PTP derived from dietary *P. microphylla* Turcz leaves as a safe supplement to vegetables for alleviating lipid metabolism disorders [45]. Additionally, the histological examination also suggested that PTP could prevent obesity by reducing the fat accumulation in the liver and decreasing the size of the tested mouse adipocytes (Figure 3). Surprisingly, the application of PTP in the HFD-fed mice significantly reduced the aberrant serum lipid metabolism in the HFD-fed mice. Previous studies have found that HDL cholesterol-induced abnormalities in lipid metabolism are related to decreased serum ADPN as well as elevated serum LPS [46]. ADPN is associated with the control of energy homeostasis, lipid metabolism, and obesity, which protects against excessive hepatic lipid accumulation, insulin resistance, and inflammation [47,48]. LPS is an endotoxin, a constituent of the cell wall of Gram-negative bacteria, and the elevated levels of circulating LPS in obese mice are a consequence of nutrient overloads causing intestinal barrier damage [49]. In our study, HFD feeding resulted in abnormal levels of ADPN and endotoxin LPS in mice, while PTP significantly antagonized the decline in the serum concentration of ADPN and the elevation of LPS in HFD-fed mice (Figure 4). These results suggest that PTP was able to produce preventive effects on dyslipidemia and hepatic steatosis caused by HFD consumption in mice.

It is well known that obesity is always accompanied by destructive glucose homeostasis and insulin sensitivity, and insulin resistance is intimately related to altered lipid profiles [50]. Consistent with previous findings, our findings suggest that the HFD generated severe glucose homeostasis abnormalities in mice, including glucose intolerance and insulin resistance (Figure 5). Interestingly, the treatment of PTP at a high dosage could dramatically enhance the insulin sensitivity in HFD-fed obese mice, which was salutary to the prevention of hyperlipidemia. This is in accordance with other studies regarding the idea that natural plant extracts may ameliorate hyperglycemia and insulin sensitivity [51,52]. PTP as the natural pectin derived from *P. microphylla* Turcz leaves was approved to exert an important biological role in maintaining glucose homeostasis.

Obesity and related metabolic disorders are primarily caused by an imbalance between energy intake and energy expenditure [8,9], and aiming for an increase in energy expenditure has therefore emerged as the most attractive strategy against obesity. Our study involved a preliminary investigation and assessment of UCP1 expression as a key regulator of fat thermogenesis in beige adipose tissue and BAT, in which the crucial thermogenic fats used elevated levels of UCP1 in the mitochondria to decouple respiration and release chemical energy as heat [8,9,53]. Although the underlying mechanisms remain unclear, phytochemicals from dietary resources (i.e., grape seed proanthocyanidins) may promote beige adipose formation and activate BAT functionality [54]. In line with earlier studies, the current study found that the HFD in mice dramatically decreased UCP1 expression in BAT. However, the expression of UCP1 in BAT was elevated and the browning transformation of subcutaneous adipose tissues was activated by the administration of PTP (Figure 6), indicating that the protective effects of PTP on obesity might be closely associated with increased energy expenditure, as evidenced by upregulating the UCP1 expression in BAT and the browning of white adipose tissue in PTP-treated mice.

Recent research has shown that HFD intake has a significant impact on the makeup of gut flora [10,11]. Gut microbiota disorders can lead to a variety of diseases, including impaired lipid metabolism and liver steatosis, as well as glucose metabolism disorders. Mounting evidence has suggested that gut microbiota play an essential part in resisting obesity and its related metabolic disorders [55,56,57]. In parallel with these reports [8,9], our study demonstrated that HFD feeding could significantly change the composition of intestinal microbiota in in comparison with untreated ND-fed mice, but administering PTP at 400 mg/kg bw efficiently reversed the HFD-induced gut microbiota dysbiosis (Figure 7). Interestingly, the treatment of PTP at a high dosage had a significant effect on the downregulation of *Firmicutes* and the increase in *Bacteroides* in HFD-fed mice (Figure 7), suggesting that PTP use may relieve imbalances in the intestinal flora of mice at the phylum level. Additionally, long-term ingestion of an HFD increased the enrichment of some possible harmful bacteria such as *Faecalibaculum*, *Romboutsia*, and *Enterorhabdus*, but reduced the abundance of some beneficial bacteria [45,46,58]. Intriguingly, compared with HFD-fed mice, PTP treatment significantly reduced the colon microorganisms, such as genera *Faecalibaculum*, *Romboutsia*, and *Enterorhabdus*, and increased the genera *norank-f-Muribaculaceae* (Figure 8). In addition, *norank-f-Muribaculaceae* was found to promote lean body mass and metabolic health in one study [59]. Interestingly, in the present study, *norank-f-Muribaculaceae* was strongly negatively correlated with obesity-related metrics, whereas treatment with PTP significantly increased its relative abundance. Therefore, we believe that changes in the intestinal microbes are an important cause of obesity, and hypothesize that PTP modifies the intestinal microbial composition by reducing the abundance of obesity-associated bacteria and enhancing the abundance of beneficial bacteria, thereby reducing the generation of obesity. Our results are consistent with previous reports that pectin can achieve obesity prevention by altering the composition of the intestinal microbes [16,20]. The quantity–effect relationship and time–effect relationship between PTP and its effects in preventing obesity needs to be further revealed in depth [11,12].

In summary, the present study demonstrates that PTP as natural pectin can prevent HFD-induced obesity by increasing BAT activity and beige fat formation, and this effect could be attributed to PTP regulation of the gut microbiota and mouse lipid metabolism. Importantly, this finding provides the possibility of utilizing PTP as a novel pectin for both the prevention and treatment of palliative chronic diseases, establishing a scientific foundation for the use of natural pectins for health benefits.

## Data Availability

The original contributions presented in the study are included in the article, further inquiries can be directed to the corresponding author.
